# Mammalian tissues defective in nonsense-mediated mRNA decay display highly aberrant splicing patterns

**DOI:** 10.1186/gb-2012-13-5-r35

**Published:** 2012-05-24

**Authors:** Joachim Weischenfeldt, Johannes Waage, Geng Tian, Jing Zhao, Inge Damgaard, Janus Schou Jakobsen, Karsten Kristiansen, Anders Krogh, Jun Wang, Bo T Porse

**Affiliations:** 1 The Finsen Laboratory, Rigshospitalet, Faculty of Health Sciences, University of Copenhagen, DK2200 Copenhagen, Denmark; 2 Biotech Research and Innovation Centre (BRIC), University of Copenhagen, DK-2200 Copenhagen, Denmark; 3 Section for Gene Therapy Research, Rigshospitalet, University of Copenhagen, DK-2100 Copenhagen, Denmark; 4 The Bioinformatics Centre, University of Copenhagen, DK-2200, Copenhagen, Denmark; 5 BGI-Shenzhen, Shenzhen 518083, China; 6 Department of Biology, University of Copenhagen, DK-2200 Copenhagen, Denmark

## Abstract

**Background:**

Nonsense-mediated mRNA decay (NMD) affects the outcome of alternative splicing by degrading mRNA isoforms with premature termination codons. Splicing regulators constitute important NMD targets; however, the extent to which loss of NMD causes extensive deregulation of alternative splicing has not previously been assayed in a global, unbiased manner. Here, we combine mouse genetics and RNA-seq to provide the first *in vivo *analysis of the global impact of NMD on splicing patterns in two primary mouse tissues ablated for the NMD factor UPF2.

**Results:**

We developed a bioinformatic pipeline that maps RNA-seq data to a combinatorial exon database, predicts NMD-susceptibility for mRNA isoforms and calculates the distribution of major splice isoform classes. We present a catalog of NMD-regulated alternative splicing events, showing that isoforms of 30% of all expressed genes are upregulated in NMD-deficient cells and that NMD targets all major splicing classes. Importantly, NMD-dependent effects are not restricted to premature termination codon+ isoforms but also involve an abundance of splicing events that do not generate premature termination codons. Supporting their functional importance, the latter events are associated with high intronic conservation.

**Conclusions:**

Our data demonstrate that NMD regulates alternative splicing outcomes through an intricate web of splicing regulators and that its loss leads to the deregulation of a panoply of splicing events, providing novel insights into its role in core- and tissue-specific regulation of gene expression. Thus, our study extends the importance of NMD from an mRNA quality pathway to a regulator of several layers of gene expression.

## Background

Alternative splicing (AS) involves the selective inclusion and exclusion of exons from a nascent pre-mRNA that results in various combinations of mature mRNAs with different coding potential and thus protein sequence [1]. Importantly, it has recently been estimated that nearly 95% of all multi-exon genes in the mammalian cell undergo AS [2,3], suggesting a pivotal role for AS in regulating and expanding the repertoire of isoforms expressed. By examining ESTs, it has been proposed that one-third of all AS isoforms contain a premature termination codon (PTC) [4], and these are expected to be targeted for degradation by nonsense-mediated mRNA decay (NMD). NMD is an mRNA quality control mechanism, and the primary function of NMD was initially thought to be in removal of aberrant transcripts arising from mutations or faulty transcription, mRNA processing or translation, but it is now evident that NMD impacts on both diverse physiological processes [5-7] as well as pathophysiological conditions (reviewed in [8]). The conserved core components of the NMD pathway are the UPF1, UPF2 and UPF3A/B proteins, and mutations or depletion of these factors inactivate NMD [9,10]. In mammalian cells, PTCs are distinguished from normal stop codons by their position relative to a downstream exon-exon junction, which is marked by the deposition of the exon junction complex [11]. It has been generally established that for a stop codon to be recognized by the NMD apparatus, it must be situated at least 50 nucleotides upstream of an exon-exon boundary (the 50 nucleotides rule) [12]. Thus, nearly all naturally occurring eukaryotic stop codons are found downstream of the last intron, thereby rendering them immune to NMD. Although recent data have demonstrated that the proximity of the poly(A)-binding protein (PABP) to the PTC is inversely correlated with the efficiency of NMD [13,14], the 50 nucleotides rule applies to almost all studied mammalian transcripts, taking heed of a few noted exceptions [15,16]. Mechanistically, AS can utilize NMD to selectively degrade transcripts by the selective inclusion of a PTC-containing (PTC+) exon or exclusion of an exon, resulting in a PTC+ downstream exon. This coupling, initially discovered for serine/arginine-rich (SR) proteins in *Caenorhabditis elegans *[17], has been coined regulated unproductive splicing and translation (RUST) or AS coupled to NMD (AS-NMD) [4,18]. Intriguingly, proteins involved in splicing processes utilize AS-NMD to autoregulate their own synthesis through a negative feedback loop. The most well characterized splicing activators, the SR proteins, bind to *cis *elements in the pre-mRNA, usually stimulating the inclusion of an exon. The SR proteins have been shown to utilize AS-NMD in a negative feedback loop to activate the inclusion of a PTC+ exon (PTC upon inclusion) in their own pre-mRNA, thus resulting in NMD [18-21]. The other major class of splice regulators, the heterogeneous nuclear ribonucleoproteins (hnRNPs), are a class of RNA binding proteins with roles in mRNA splicing, export and translation [22,23]. The hnRNPs often, but not always, bind to splice silencer elements and repress splicing at nearby splice sites. Splicing repressors, such as hnRNPs, use AS-NMD to repress the inclusion of a coding exon in their own pre-mRNA that leads to an out-of-frame skipping event, consequently inducing a downstream PTC and thus NMD (PTC upon exclusion). Moreover, AS-NMD is also used to cross-regulate expression of other splice factors, as described elegantly for PTBP1 and PTBP2 [24].

AS is regulated by the selective recruitment of splice regulators to pre-mRNAs. It is well established that splicing activators (such as SR proteins) compete with splicing repressors (such as hnRNPs) for binding to splice sites in an antagonistic manner, where the relative concentration of the two classes regulates the level of AS [25,26]. Thus, the fate of an alternative exon is usually decided by the antagonism between SR proteins and hnRNPs and their concentration and activity (reviewed in [27]). Due to the autoregulatory feedback loop employed by splice regulators, modulating NMD could potentially have widespread effects on the concentration of splicing activators and repressors and thus AS and AS-NMD.

Despite the potential of AS-NMD, it is presently not known to what extent this pathway regulates the transcriptome on a global scale, and how AS homeostasis is affected by NMD. A major problem in discovering the full spectrum of PTC+ transcripts is that EST and cDNA repositories are biased against these isoforms due to their unstable nature in normal cells. Additionally, most studies have primarily focused on microarrays to query the transcriptome upon muting NMD [18,19,28], and the newer studies using sequencing [29] have focused on single cassette exon events, thus disregarding many other physiologically important splice classes such as mutually exclusive exons and alternative 5' and 3' splice site usage. Last but not least, the transcriptomic consequences of genetically modulating NMD and thereby AS in the mammalian organism are largely unanswered.

In the present study, we have performed RNA-seq on two different tissues from a *Upf2 *conditional knock-out (KO) mouse line. Thus, in addition to providing significantly novel insights into common and tissue-specific functions of NMD, our study represents the first comprehensive and unbiased transcriptome analysis of adult genetically modified mice. To facilitate a high-resolution analysis of all possible exon-exon combinations, we have generated a bioinformatic pipeline, named RAINMAN (RnAseq-based Isoform detection and NMd ANalysis pipeline; available to the scientific community), that maps reads to a combinatorial database that incorporates both known and *in silico *predicted exon-exon junctions. The pipeline predicts NMD susceptibility based on junction evidence and groups AS events into seven major splice isoform classes. Using this approach, our results reveal an unprecedented increase in AS upon ablating UPF2, and by inference NMD (although small additive effects from UPF3A/B interaction and deregulation of UPF1 phosphorylation cannot be wholly excluded from analysis, we juxtapose UPF2 and NMD ablation for all purposes in this work) and show that a high proportion of these upregulated AS events are not predicted to contain a PTC. Hence, we find that only 50% of the increase in AS is directly due to stabilization of PTC+ isoforms. Our data demonstrate that muting NMD results in deregulated levels of core splice regulators at both the mRNA and protein level, and further suggests that this contributes to the deregulation of general AS upon ablating NMD. Hence, our data support a model where NMD ablation leads to deregulated levels of core splicing factors that ultimately lead to aberrant global splicing, implying a novel intricate interplay between the NMD machinery and the splicing factors. Finally, our data analysis generates the first insights into a putative functional role of the PTC and its surrounding regions through analysis of their conservation patterns.

## Results

A major task in analyzing AS by RNA-seq is to explore and quantify the differential representation of various splice classes and the protein-coding potential of the mapped reads. To this end, we have developed RAINMAN, a streamlined bioinformatic pipeline available to the scientific community that maps RNA-seq reads to a comprehensive combinatorial database of exon-exon junctions for unique junction discovery, PTC detection and identification of splice isoforms. We used this pipeline to investigate the complexity of the transcriptome and global role of AS-NMD *in vivo *in mammalian cells, by taking advantage of our *Upf2 *conditional KO mouse, which we previously used to demonstrate the *in vivo *importance of NMD [7,30]. To explore the effect of NMD on global splicing and to generate and validate an attractive bioinformatic pipeline to study AS and NMD, we chose to analyze the transcriptomes of two different mammalian organ systems with distinct phenotypes upon UPF2 deletion. In one end of the spectrum, we analyzed liver, wherein removal of UPF2 results in failure in liver metabolism and a high mortality rate [30], and in the other, we analyzed bone marrow-derived macrophages (BMMs). These macrophages are generated *in vitro *from murine bone marrow cells, and *Upf2 *deleted BMMs are completely devoid of NMD activity but nevertheless show no morphological or functional phenotype compared to wild-type (WT) controls [7].

### Splice isoform inference and PTC detection

**Figure 1:**
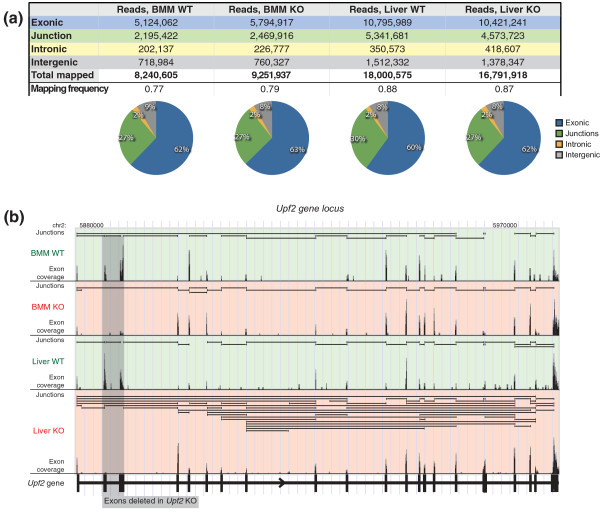
**Mapping and downstream analysis of RNA-seq data**. **(a) **Total number of RNA-seq reads mapped to exonic, junction, intronic and intergenic regions as well as the fraction of mapped reads for all four samples. Pie charts below visualize the distribution of all mapped reads. **(b) **An example of an output from the pipeline, visualized in the UCSC genome browser, showing the *Upf2 *gene locus, with junctions supported by reads (horizontal bars in each sub-window), and exon coverage (vertical bars). Refer to Figure S1 in Additional file 2 for another example of a genome browser output. No minimum read cutoff was used for junction visualization. The very low exon coverage present for knocked out exons 2 and 3 in KO samples represents a miniscule amount of non-recombined tissue/cells.

In order to obtain biological material, we first generated mice in which the NMD core factor *Upf2 *could be selectively inactivated in liver or in BMMs using our previously reported strategy (see Materials and methods). We performed whole transcriptome sequencing (single-end) on poly (A)-purified RNA, isolated from poly-IC injected *Upf2^fl/fl^; Mx1Cre *and *Upf2^fl/fl ^*livers (termed from now on 'Liver KO' and 'Liver WT', respectively) and *Upf2^fl/fl^; LysMCre *BMMs and *Upf2^fl/fl ^*BMMs (henceforth 'BMM KO' and 'BMM WT', respectively). In order to minimize biological variation, we generated libraries from pools of poly(A)-purified RNA derived from three individual animals. Due to the underrepresented nature of NMD-susceptible transcripts in EST and cDNA databases [4], we generated a comprehensive combinatorial database of exon-exon junctions in the murine genome, using sequences from exon models annotated in different repositories (RefSeq, Ensembl, UCSC Known Genes, GENSCAN and Exoniphy; Supplemental Materials in Additional file 1 and Figure S1B in Additional file 2). To discover junctions between exons not previously recorded in the murine transcriptome, we also employed TopHat [31], a *de novo *mapping algorithm, to map reads to junctions between unannotated exons (Table S1 in Additional file 1). To utilize reads that cover three or more exons, thus aligning to two or more exon-exon junctions, and not initially mapped to our combinatorial database or by TopHat, we incorporated an extra read truncation step (Figure S1B in Additional file 2), trimming reads in steps of 10 bp and remapping, allowing us to recover an additional 4 to 7% of total reads. As a result, 84% of reads mapped to the genome or transcriptome, and out of these, 28% mapped to splice junctions (Figure 1a), corresponding to a combined total of 323,474 unique splice junctions across our two tissues and two genotypes. With a minimum requirement of three reads to a splice junction, minimizing sequencing and mapping artifacts, we mapped approximately 150,000 unique junctions. Of note, we found that the *de novo *mapper TopHat contributed significantly to our transcriptome set, with 11 to 16% of all discovered junctions uniquely defined by TopHat (Table S1 in Additional file 1). Mapping of the KO samples benefited the most from these TopHat predicted splice junctions, suggesting that the curated transcript repositories are biased against the TopHat-predicted junctions due to their NMD susceptibility. Indeed, TopHat identified 20% and 24% of the PTC+ splice junctions above our minimum read cutoff, compared to only 4% and 11% of PTC- splice junctions (junctions that do result in the generation of a PTC, in BMM and liver samples, respectively (Table S2 in Additional file 1). These data demonstrate that at least a quarter of NMD susceptible transcripts are not present in the normal murine repositories. This has implications for future splice isoform detection, since many transcripts that are below detectable levels under physiological conditions, and hence are absent in the repositories, will not be detected under conditions that could favor their presence, for example, perturbed or disease states. Thus, taking advantage of our combinatorial database that includes a pipeline for splice isoform detection and PTC prediction is likely to considerably increase the number of isoforms detected under conditions that favor increased AS.

In the next step of RAINMAN, the pipeline calculates the distribution of seven different splice isoform classes and predicts PTCs (see Supplementary Methods in Additional file 1 for details), and all data are combined for easy visualization and data mining. An illustrative example of a genome browser output from the mapping steps is shown in Figure 1b, with the conditional *Upf2 *KO gene deleted for exons 2 and 3 in the KO sample. The UCSC-based visualization includes both evidence of reads mapped to junctions and to exons, and, in this case, demonstrates increased AS in the liver KO compared to WT.

**Figure 2:**
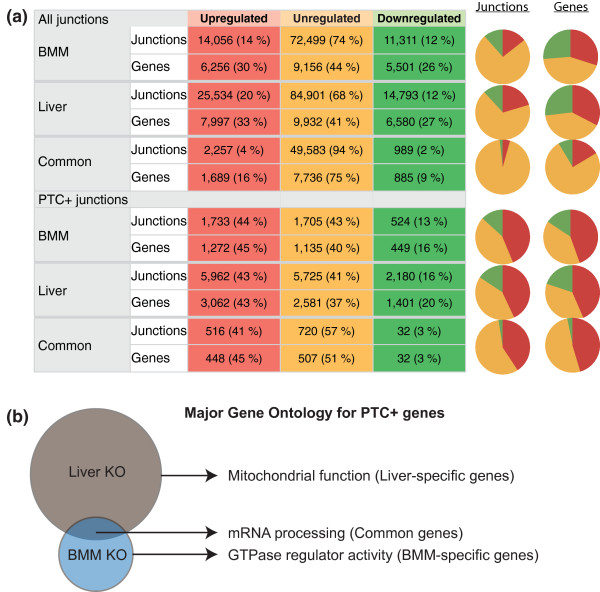
**Number of regulated splicing and PTC occurrences in *Upf2 *KO tissues**. **(a) **Splice junctions with a minimum of three reads in WT or KO samples were grouped into upregulated (≥2-fold up in KO), downregulated (≥2-fold down in KO) or unregulated. Genes corresponding to the junctions are also shown. Left: number of junctions and genes and percentage in parentheses for the relative fraction in each tissue. The top half of the table lists results for all junctions, the bottom half for PTC+ junctions only. Right: pie charts visualize the relative distribution of regulated junctions and corresponding genes. **(b) **Venn diagram of major Gene Ontology terms associated with liver-unique genes (top), BMM-unique genes (bottom) and common genes (overlap).

We mapped a total of 150,000 unique junctions with approximately 100,000 and 130,000 junctions in BMM and liver tissues, respectively (minimum of three reads to a junction; Figure 2a, top, and data not shown). Tallying up, we found that 6,256 and 7,997 unique genes harbored 14,056 and 25,534 upregulated junction events in BMM and liver, respectively, an average of 2.3 and 3.2 upregulated junction events per gene (Figure 2a), demonstrating that loss of UPF2, and by inference NMD, leads to a substantial deregulation of splicing.

### Ablating NMD results in significant upregulation of PTC+ junctions

It has been shown that a stop codon is generally recognized as a PTC by the NMD machinery, if it is situated at least 50 nucleotides upstream of an exon-exon junction, termed the 50 nucleotides rule. We assayed and verified this distance requirement by simply plotting the position of RefSeq gene model stop codons that reside in the penultimate exon (Figure S2 in Additional file 3), observing that the vast majority of these stops indeed fall precisely within 50 nucleotides of the final intron boundary, and thereby avoiding the elicitation of NMD for those transcripts. Incorporating this distance metric in our PTC prediction algorithm, we calculated the fraction of regulated PTC+ junctions as a function of UPF2 ablation. As expected, this analysis demonstrated a highly significant upregulation of PTC+ junctions in the KO samples (Figure 2a, all junctions versus PTC+ junctions, *P-*value 1 × 10^-16^, Chi-square). In total, approximately 44% of predicted PTC+ junctions were upregulated in BMMs and Liver KO (Figure 2a), amounting to 16% (BMM) and 28% (liver) of expressed genes containing at least one NMD-susceptible splice junction regulated more than two-fold.

Among the upregulated PTC+ splice junctions, 516 were shared between the two tissues, and these corresponded to 448 unique genes (Figure 2a; Table S3 in Additional file 4), which we thus termed core NMD targets. The group includes well-known NMD targets such as *Smg5*, *Hsf1*, and *Zcchc6 *as well as many known NMD-susceptible splicing factors [6,18,19]. Indeed, Gene Ontology (GO) analysis demonstrated that the highest ranked cluster contained genes involved in mRNA processing and splicing (*P*-value of 9.0 × 10^-18^, Bonferroni corrected; see the full list of GO terms and genes in Table S3 in Additional file 4). All classical SR proteins have been shown to utilize AS-NMD to autoregulate their own synthesis [20], and our finding that common splicing factor junctions are upregulated upon muting NMD is in agreement with earlier studies [7,18,19,32]. We also determined the GO terms associated with genes containing upregulated PTC+ junctions unique to either liver or BMMs (Figure 2b). In the BMM-specific set, genes involved in G-protein coupled receptor function were enriched (*P*-value 1.5 × 10^-4^, Bonferroni corrected; Table S4 in Additional file 5), whereas the liver specific set was strongly associated with mitochondrion, among others (*P*-value 9.7 × 10^-56^, Bonferroni corrected; Table S4 in Additional file 5).

In summary, we find that 43 to 44% of all predicted PTC+ junctions are upregulated in *Upf2*-ablated tissues and that 516 of these junctions are common in BMM and liver, several of which are well-known NMD targets. Moreover, we show that NMD is predicted to downregulate isoforms of 16 to 28% of all expressed genes (excluding the well-described regulation of splicing factors from the analysis). Moreover, NMD regulates genes involved in mitochondria and G-protein-coupled receptor functions in liver and BMM, respectively, suggesting that NMD also serves tissue-specific functions.

### Loss of NMD leads to the selective stabilization of alternative splicing events

**Figure 3:**
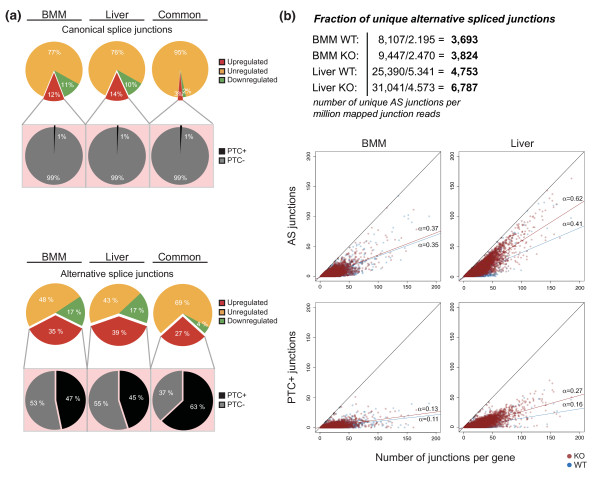
**Loss of UPF2 leads to increased AS-NMD**. **(a) **Proportions of regulated canonical junctions versus junctions supporting AS events (red, upregulated; yellow, unregulated; green, downregulated), with additional distribution pie charts underneath displaying the predicted percentage of PTC+ junctions versus PTC- junctions (junctions that do not elicit a PTC) of upregulated junctions (black, PTC+; grey, PTC-). **(b) **The number of AS junctions and the number of PTC+ junctions are shown as a function of number of junctions per gene in the top and bottom panels, respectively. The slopes of the curves were calculated by linear interpolation. In total, our pipeline detected 10,061 unique AS junctions in BMMs (8,107 in WT and 9,447 in KO) compared to 33,164 in the liver (25,390 in WT and 31,041 in KO), and calculations in the table (top) show the number of unique AS junctions per million mapped reads for each sample. No minimum read cutoff was used in (b).

We next compared the impact of ablating NMD on canonical versus AS in more detail. Here, a canonical junction is defined as a splicing junction between two consecutive exons of the longest RefSeq isoform for each gene, and AS junctions are thus all other combinations. In total, our pipeline detected approximately 10,000 unique AS junctions in BMMs compared to approximately 35,000 in the liver (Figure 3). A significant challenge in analyzing expression changes upon NMD ablation is to distinguish primary from secondary effects. Using the sensitive PTC-detection in RAINMAN, we found that close to 50% of AS was predicted to generate a PTC (Figure 3a, bottom), which is in stark contrast to canonical splicing junctions (Figure 3a, top). This suggests that NMD directly degrades 16% (0.47 × 0.35) of all AS in BMMs and 18% in liver (0.45 × 0.39), resulting in more than a two-fold reduction of the involved isoforms.

The BMM and liver samples demonstrated essentially similar fractions of up- and downregulated AS junctions (Figure 3a). However, the number of unique AS junctions were higher in liver samples, even after calibrating for the number of mapped junction reads (and thereby sequencing depth; Figure 3b, top). To investigate whether this was a characteristic feature of the organs or whether it was due primarily to muting of NMD, we analyzed the AS complexity per gene (Figure 3b). The number of mapped junction reads was first normalized to BMM WT (Figure 1a) by stochastically removing reads from BMM KO, liver WT and liver KO, to exclude any skewing due to differences in sequencing depth. Interestingly, these data demonstrate an inherently different AS profile between the two tissues. Whereas BMM WT and KO showed similar proportions of AS, measured as the correlation between the number of AS junctions and the total number of junctions per gene (Figure 3b, top), the proportion of AS of the liver KO sample increased considerably more compared to both BMM KO and liver WT (Figure 3b, top). This strongly suggests that the liver has a high level of AS relative to BMM, both normally and through ablation of NMD.

Finally, we also compared the proportion of PTC+ junctions, measured as the number of PTC+ junctions as a function of total splicing for each gene (Figure 3b, bottom). The PTC+ junctions followed the same trend as for AS junctions, with a steeper relative increase in PTC+ junctions in liver KO compared to liver WT and BMM. From linear interpolation, we found that, on average, 27% of all spliced junctions in a given gene in the liver are predicted to result in a PTC (liver KO slope of 0.27; Figure 3b).

In conclusion, these data demonstrate that approximately 17% of all AS is downregulated via NMD more than two-fold. We furthermore show that liver is characterized by a high degree of AS compared to BMMs and that close to one-third of all uniquely spliced junctions in the liver are predicted to elicit a PTC, thus giving the first *in vivo *evidence of the pervasive effect and importance of AS-NMD in the mammalian organism.

### Splice isoform classes are differently affected by NMD ablation

**Figure 4:**
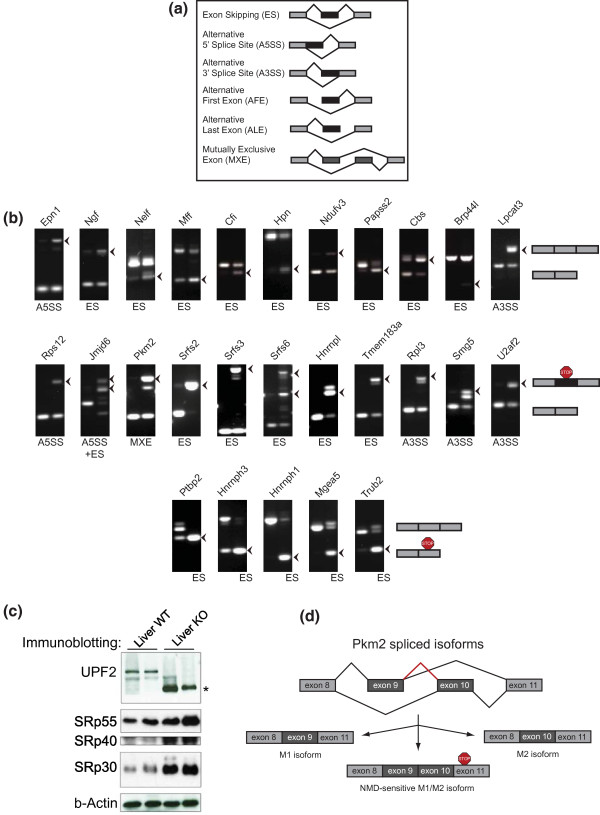
**Splice isoform classes are differentially affected by loss of UPF2**. **(a) **Schematic drawing of the main isoform classes detected in our pipeline. **(b) **RT-PCR validation of 25 splicing events predicted from our pipeline (see Table S9 in Additional file 12 for a list of the 49 validated events out of 50 tested). Top: normal AS events. Middle: PTC upon inclusion events. Bottom: PTC upon exclusion by ES. **(c) **Western blotting from two different liver pairs, showing UPF2 (rabbit α-UPF2), SRp55, SRp40 and SRp30 (mouse α-mAb104) and β-actin (rabbit α-actin). The asterisk denotes the truncated UPF2 isoform found in cells ablated for NMD. **(d) **MXE splicing of Pkm2 and the inclusion of both mutually exclusive exons in the KO sample.

As described above, we detected a significant increase in AS upon ablating UPF2. It has, however, not previously been described to what extent NMD affects different splicing classes. Transcriptome-wide studies where NMD has been muted have primarily looked at single cassette exon skipping (ES) events [28,32], thus disregarding many physiologically relevant AS events. To this end, we implemented an AS isoform classification module that allowed us to infer the degree to which all major splice isoform classes were affected by NMD. Hence, our splice isoform inference pipeline assessed the distribution of splice junctions to seven major groups of AS events, namely single exon skipping (SES) and multiple exon skipping (MES), alternative 5' splice site (A5SS) and alternative 3' splice site (A3SS), mutually exclusive exons (MXE), alternative first exon (AFE) and alternative last exon (ALE) (see Table 1, Supplementary Materials in Additional file 1 and Table S6 in Additional file 6 for pipeline performance). We narrowed our downstream splice isoform analysis to those that demonstrated a change in 'percent spliced in' (PSI) between KO and WT (ΔPSI) higher than 20%. PSI is calculated from the ratio of junctions supporting a given feature (for example, the inclusion of an exon) versus junctions supporting the reciprocal event (for example, the skipping of the same exon), and the ΔPSI is thus a metric of how much the inclusion changes upon NMD ablation (see Supplementary Methods in Additional file 1 for further details). We found that approximately 40% of all ES events with a ΔPSI higher than 20% (inclusion) or lower than -20% (exclusion) in the KO tissues were upregulated due to stabilization of a PTC+ isoform (Table 1). Next, we subdivided ES events into PTC upon inclusion and exclusion events. An example of a PTC upon exclusion event that is stabilized upon NMD ablation in both BMM (ΔPSI = -61%) and liver (ΔPSI = -63%) is *Mgea5*, which was also found upregulated in immortalized mouse embryonic fibroblasts (MEFs) ablated for NMD [32]. We validated this *Mgea5 *isoform among others by RT-PCR (Figure 4b). In the liver, 55% of all single exon inclusion events generated a PTC, whereas 40% were found in the BMM (compare PTC upon inclusion to total inclusion events for single ES in Table 1). *Tmem183a *is an example of a highly skipped PTC upon inclusion that was found stabilized in both BMM (ΔPSI = 51%), liver (ΔPSI = 54%) and immortalized MEFs ablated for NMD [32], and was similarly validated by RT-PCR (Figure 4b).

**Table 1:** Splice events and PTC+ classification

Tissue	Splice event	Total events	PTC+ events	Percentage PTC+	Exclusion events	PTC upon exclusion	Inclusion events	PTC upon inclusion
BMM	Total ES	730	281	38%	428	201	302	80
	Single ES	511			310	148	201	80
	Multiple ES	219			118	53	101	-
	A5SS	130	68	52%				
	A3SS	238	110	46%				
	MXE	44	5	11%				
	AFE	130	44	34%				
	ALE	49	NA	NA				
								
Liver	Total ES	3,102	1,285	41%	1,926	965	1,176	320
	Single ES	1,505			932	531	573	320
	Multiple ES	1,597			994	434	603	-
	A5SS	449	263	59%				
	A3SS	654	370	57%				
	MXE	475	66	14%				
	AFE	172	66	38%				
	ALE	143	NA	NA				

Splice isoform classes are shown for both single and multiple exon skipping (ES), alternative 5' splice site (A5SS), alternative 3' splice site (A3SS), mutually exclusive exons (MXE), alternative first exon (AFE) and alternative last exon (ALE). For ALE, no events were classified as PTC+, since the class relies on splicing to two different last exons. Here, a ΔPSI > 20% was required for all classes. NA, not applicable.

Interestingly, for alternative usage of A5SSs and A3SSs, we saw an even higher proportion of PTC+ events, especially in the liver (compare the fourth and fifth rows in BMM versus liver tissue in Table 1), suggesting a previously unrecognized importance for these splice events in AS-NMD. The ribosomal protein *Rps12 *is an example of a gene that generates a PTC+ isoform as a result of A5SS in both BMM (ΔPSI = 50%) and liver (ΔPSI = 55%), and likewise for *Smg5 *in BMM (ΔPSI = 37%) and liver (ΔPSI = 50%) as a result of A3SS (Table 1).

Using stringent criteria (Supplementary Methods in Additional file 1), we detected 44 MXE events in the BMM KO, but more than 10 times as many in the more splice-prone liver KO tissue (Table 1). Out of the detected MXE events, few were predicted to elicit a PTC+ isoform, possibly due to the regulated nature of a mutually exclusive splice event, which in most instances would not be predicted to undergo NMD. An exception is the pyruvate kinase gene *Pkm2*, which encodes two different enzymatic active isoforms due to a single MXE event [33]. In the KO samples, and this was particularly true for BMMs, aberrant splicing resulted in inclusion of both mutually exclusive exons, resulting in a PTC+ isoform (validation in Figure 4b and schematic in Figure 4d). This may indicate an important role for NMD in ensuring the exclusive incorporation of exons in MXE events.

Finally, we quantified the number of isoforms with an increase in AFE or ALE. From transcriptome data alone, it is often impossible to determine whether an AFE is the result of alternative promoter usage or AS between two mutually exclusive first exons and the second exon. Nevertheless, AFE results in alternative transcripts with potential PTCs. Analysis of AFE showed that more than one-third of the detected AFE events are predicted to generate a PTC+ isoform (Table 1). For ALE categorization, we required AS to two mutually exclusive 3' terminal exons, and PTC+ prediction is thus not meaningful. Hence, changes in levels of ALE are therefore most likely secondary effects of altered splicing upon UPF2 ablation. A functional mechanism for ALE is the regulated inclusion of microRNA target sites in the 3' UTR, and this potentially adds another layer of complexity when studying conditions where splicing is perturbed, such as by removing NMD.

To test the accuracy of our splice isoform detection algorithm, we chose 50 RAINMAN-predicted AS events (ΔPSI > 20% (inclusion), or < -20% (exclusion)) for RT-PCR on independent liver and BMM material. We validated AS events that are not predicted to result in a PTC+ isoform (Figure 4b, top row), isoforms stabilized in the KO due to inclusion of a PTC (Figure 4b, middle row) and isoforms that elicit a PTC due to a skipping event (Figure 4b, bottom row). Out of the 50 tested AS events, we were able to validate 49 of these (98%), and we therefore conclude that our splice isoform detection algorithm is highly accurate in predicting different splice isoform events.

To validate the inferred expression changes and to assess inter sample-replicate variability, we performed quantitative PCR experiments on biological replicates. This analysis showed a strong correlation with the RNA-seq inferred expression changes (R = 0.975, Pearson's correlation; Figure S3A in Additional file 7) and a very low sample replicate variability (Figure S3B in Additional file 7). In addition, we assessed our ability to correctly infer PTC+ isoforms by cloning and sequencing the full-length isoforms of *Srsf9*, a gene with a PTC-upon inclusion event (ΔPSI = -40% in liver; isoform not detectable in BMMs). The predicted PTC+ isoform was precisely recapitulated *in vivo *(Figure S3C in Additional file 7), further validating the usefulness of our approach.

### Loss of NMD leads to deregulation of PTC- isoforms through breakdown of splice factor homeostasis

We have demonstrated that only 50% of upregulated AS junctions are predicted to be the direct result of stabilized PTC+ isoforms, suggesting that a major proportion of the increased AS is the indirect result of perturbed NMD (Figure 3a). As we have shown here, the liver has a high degree of AS (Figures 1 to 3), and it has been found to have a prominent divergent expression pattern of SR proteins and hnRNPs compared to other mammalian tissues [34]. Hence, we hypothesize that this could make the liver particularly susceptible to changes in splicing patterns upon ablation of NMD.

Splicing factors are known to utilize AS-NMD to autoregulate their own synthesis in a negative feedback loop, through the selective inclusion or exclusion of PTC+ exons [4,18]. Indeed, our RNA-seq analysis also identifies a range of these factors among the core NMD targets (Figure 2; Table S3 in Additional file 4). However, as most of the NMD sensitive splicing factor isoforms are predicted to encode truncated proteins, the rescue of these upon NMD ablation is not expected to affect AS *per se *(unless they encode proteins with dominant negative properties). Previous microarray-based studies have not addressed the formal possibility that the stabilization of PTC+ isoforms upon loss of NMD could occur against the backdrop of changed levels of the canonical isoforms capable of encoding the full-length form of proteins such as splicing regulators. To test this possibility, we scrutinized our datasets for changes in the expression levels of 25 canonical mRNA isoforms capable of encoding full-length splicing regulators by correcting the changes in the gene expression ratios between KO and WT samples with the fraction contributed by the PTC+ isoforms (Table S5 in Additional file 1). Strikingly, for the 19 splice factors for which we have evidence for AS-NMD in the liver, 10 displayed a > 1.5-fold deregulation, with 5 displaying increased (*Sfrs3*, *Srfs4*, *Tra2a*, *Srfs16*, *Hnrpdl*) and 5 decreased (*Tra2b*, *Hnrnpf*, *Hnrnph3*, *Hnrnpr*, *Ptbp2*) expression of the canonical mRNA isoforms. Moreover, western blot analysis revealed that SRp30 and SRp40 protein levels were elevated in KO liver, demonstrating that the changes in canonical mRNA isoform levels for at least some splicing factors are indeed translated into full-length protein.

Collectively, these findings support a model where the increase in global AS upon loss of NMD is at least in part caused by a massive deregulation of key splicing factors, and further help to explain the PTC- AS fraction deregulated upon muting NMD. A highly aberrant level of splice regulators would likely tilt the normal balance between activators and repressors and deregulate a large subset of exons [4,18].

### Ablating NMD leads to skipping of highly conserved cassette exons

**Figure 5:**
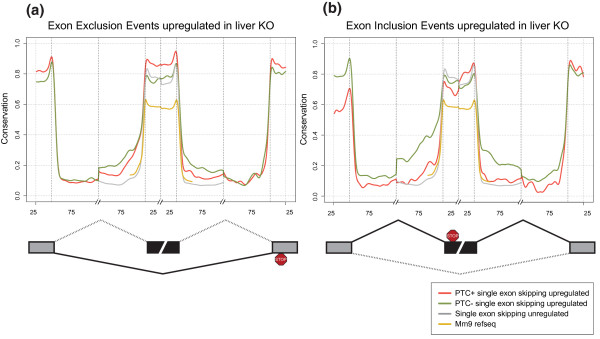
**Introns flanking regulated exons are highly conserved**. **(a, b) **Mean per position phastCon conservation scores around SES events are shown for exclusion events upregulated in the KO sample (a), and for inclusion events upregulated in the KO sample (b). Shown are PTC+ exclusion/inclusion events (red line), PTC- exclusion/inclusion events (green line) and unregulated skipping events (grey line). Yellow lines are scores for all mm9 RefSeq exons and 75 bp into surrounding introns. Data shown are for liver. Exclusion events: PTC+, 439; PTC-, 251. Inclusion events: PTC+, 64; PTC-, 162. Unregulated events: 3,494. Mm9 RefSeq exons: 274,281. See Figure S4 in Additional file 10 for a graph with BMM data. Numbers on the x-axis indicate nucleotide intervals - 25 and 75 nucleotides for exons and flanking introns, respectively. Curves represent a cubic smoothing spline fitted to data.

SES represents the best characterized AS event, and is a frequent PTC-generating mechanism. RAINMAN classified 40% and 60% of all SES as PTC+ events in the BMM and liver KO samples (Table 1 - compare PTC upon exclusion with total exclusion events, and PTC upon inclusion with total inclusion events). Out of the PTC upon inclusion events, a total of 26 events were upregulated in both KO tissues (ΔPSI > 20%) and 51 PTC upon exclusion events were similarly upregulated in both KO tissues (ΔPSI < -20%; manually curated lists are included in Table S7 in Additional file 8 and Table S8 in Additional file 9). In support of an important physiological function, almost all of the genes harboring these highly upregulated PTC+ isoforms were also upregulated at the gene level in KO samples (Table S7 in Additional file 8 and Table S8 in Additional file 9), suggesting that stabilization of the PTC+ isoforms led to an appreciable increase in total mRNA levels in KO tissues. It should be noted that most of the splicing factors and ribosomal proteins known to use AS-NMD were indeed upregulated in both tissues, but had ΔPSI > -20% or < 20% in BMMs (data not shown). Hence, the high frequency of PTC+ splice events suggests that these are regulated AS events. It is generally found that AS exons are more conserved than constitutive exons, especially in the flanking introns [35]. The core members of the SR protein family and hnRNPs have been proposed to use AS-NMD in an autoregulatory loop, by binding to highly conserved regions, termed ultraconserved elements (UCEs), to elicit the PTC+ splice event [18,20]. Upon inhibition of NMD in cell lines, it has been shown that many PTC+ exons are surrounded by high intronic conservation [18,19]. As described above, our sensitive approach demonstrated a high proportion of PTC- AS events upregulated in KO tissues, and we therefore examined the conservation of PTC+ and PTC- single exon exclusion (ΔPSI < -20%) and inclusion (ΔPSI > 20%) events in the KO samples compared to the unregulated skipping events. The latter control group contains exons that undergo AS but are not differently skipped between WT and KO (-20% < ΔPSI ≤ 20%). We first examined exclusion events, and found that flanking introns of the skipped exons are significantly more conserved compared to unregulated skipping events (Figure 5a; *P*-value < 2.2 × 10^-16^, Komogorov-Smirnov test), and this was even more pronounced in BMMs (Figure S4 in Additional file 10). Interestingly, flanking introns of PTC+ upon exclusion events were less conserved than PTC- exclusion events, but displayed a significant higher exonic conservation (P-value < 2.2 × 10^-16^, Komogorov-Smirnov test). We next considered exons that demonstrated increased inclusion in KO samples. In these cases, flanking introns were also significantly more highly conserved compared to skipping events not regulated by NMD (Figure 5b). Again, PTC- inclusion events were more highly conserved compared to PTC+ events in the flanking introns.

In summary, these results demonstrate that 40 to 60% of upregulated SES events are predicted to undergo NMD, depending on tissue type, and that these splice events are highly conserved. Importantly, PTC- inclusion and exclusion events displayed higher intronic conservation compared to PTC+ events, strongly suggesting that the UCE-containing splice regulator isoforms (which are all PTC+) are not the primary reason for the high intronic conservation surrounding skipped exons. Moreover, removal of UCE elements from the analysis did not alter the intronic conservation profiles of either PTC- or PTC+ events (data not shown). Thus, the high conservation for both included and excluded exons appears to be a general attribute of highly regulated cassette exons (ΔPSI > 20%) that are affected directly and indirectly by NMD. Thus, the deregulation of PTC- events is therefore consistent with a mechanism where deregulated levels of splice regulators in the KO samples markedly affect regulated splicing of their cognate target exons. This again implies that ablating *Upf2*, and by inference NMD, has important secondary effects on splicing factor homeostasis and that this impacts widely on global AS.

### Muting NMD leads to increase in splicing by-products

**Figure 6:**
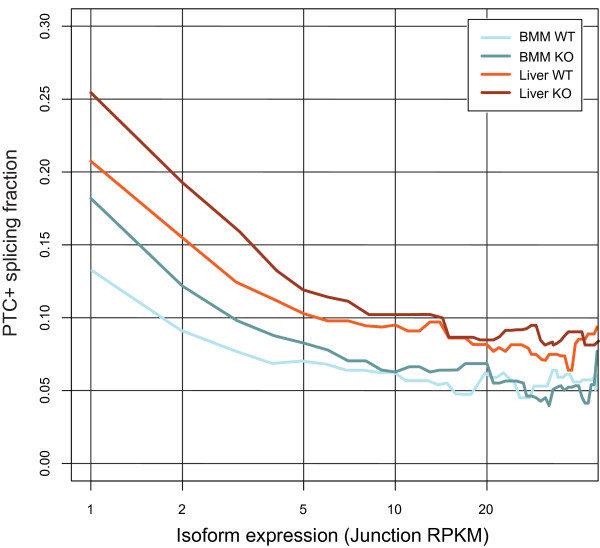
**Low-expressed junctions are enriched in *Upf2 *KO samples**. PTC-content (y-axis) in junctions binned by expression (RPKM; x-axis). All junctions for samples were binned in increments of 1 RPKM and the PTC+/PTC- ratio for each bin was calculated. No minimum read cutoff was applied.

We have demonstrated that removing UPF2, and by inference NMD, causes a dramatic increase in AS, in part by stabilizing PTC+ AS isoforms. Apart from AS-NMD, NMD has been implicated in removal of genomic noise and splice errors that might otherwise lead to a panoply of spurious isoforms [7,36-38]. The extent to which NMD destroys such splice by-products has not, however, been studied on a transcriptome-wide basis in mammalian tissues. We therefore examined the relative expression level of PTC+ junctions, measured as the PTC+ fraction (PTC-generating junctions/All other junctions) against the junction expression level (Figure 6), binned by the RNA-seq metric RPKM (reads per kb of gene model per Mb of mapped reads, refer to [39]). These data show that the PTC+ fraction is enriched in liver compared to BMM over the entire expression profile, further emphasizing that the liver is characterized by an increased abundance of PTC+ isoforms compared to BMM. Importantly, there is a distinct overrepresentation of PTC+ isoforms in the lower expression range for both liver (Figure 6 - compare dark red and light red for liver KO and WT, respectively) and BMM (Figure 6 - compare dark blue with light blue for BMM KO and WT, respectively) relative to more highly expressed isoforms. These data thus confirm and quantify, on a global scale, another major role for NMD in removing low-abundance isoforms such as the products of erroneous splicing.

### PTC exons are distinct from normal stop codon exons

**Figure 7:**
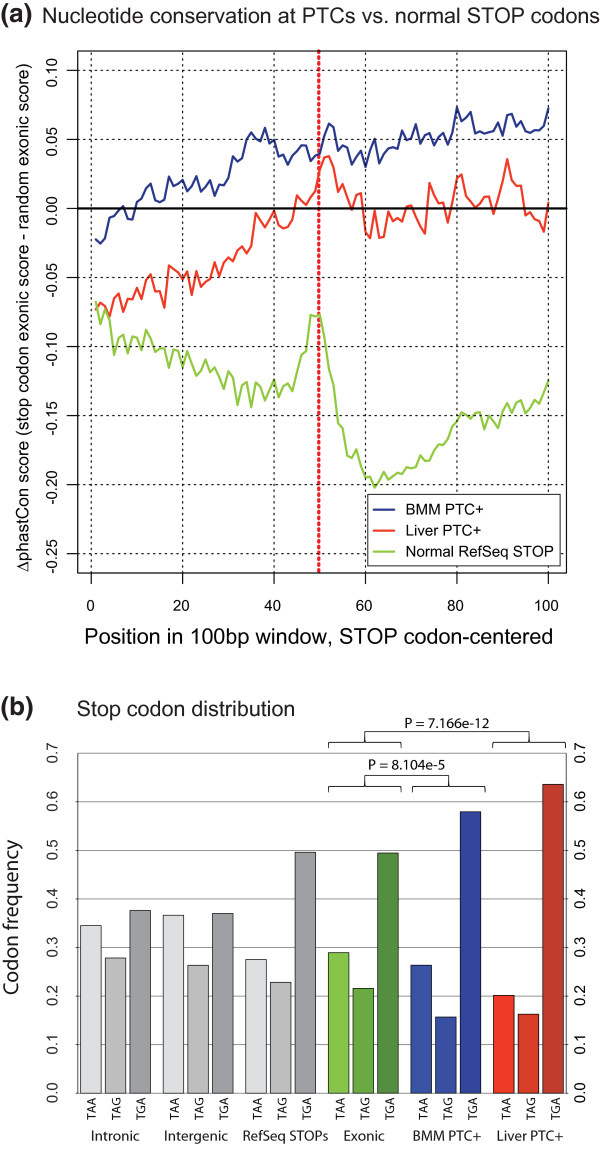
**Conservation of regulated PTCs and surrounding exons**. **(a) **Mean per-position phastCon scores are shown, centered on the PTC, for upregulated junctions in liver and BMM. To visualize conservation around PTCs in comparison to normal exonic areas, phastCon scores from a random sample (*n *= 4,000) of BMM and liver-expressed exons were subtracted from either sample. For normal STOPs, phastCon-scores from random RefSeq exons (*n *= 4,000) were subtracted. Normal STOPs are based on all RefSeq transcript models. Ranges of scores do not extend into introns, and may be shorter than 100 bp for individual PTCs. For PTC+ junctions, a KO/WT fold change of 2 was required. BMM PTC+ positions: 884. Liver PTC+ positions: 3,091. Normal RefSeq STOP positions: 23,231. **(b) **Distribution of stop codons: for intronic, intergenic and exonic bins, all mm9 trinucleotides in all three reading frames were sampled. RefSeq STOPs represent normal stop codons for all 21,470 RefSeq transcript models (for genes with multiple models, the longest was used). For BMM (*n *= 497), PTC-inducing junctions were required to have a log2(KO/WT) fold change > 2, and a minimum of 5 reads for both genotypes summed. For liver (*n *= 670), PTC-inducing junctions were required to have a log2(KO/WT) fold change > 2, and a minimum of 10 reads for both genotypes summed. Fisher's exact test was used to test for significance.

We have shown that NMD-affected SES events demonstrate a high conservation score and that this is not due to UCE-containing SR proteins and hnRNPs. The question then arises to what extent the PTC+ exon itself is aberrant compared to other expressed exons, and in particular the normal stop codon-containing exon. We thus examined the conservation of the exonic sequence surrounding the PTC in upregulated PTC+ exons, as evidenced by upregulated PTC+ junctions. To normalize for normal exonic conservation variation and for biases in tissue-specific exon conservation, we subtracted random exonic sequences expressed in the same samples. Interestingly, we found that PTC+ exons had a markedly different conservation profile compared to normal stop codon exons (Figure 7a; for comparison, see unnormalized conservation score in Figure S5 in Additional file 11). Hence, exonic sequence conservation increased towards the PTC, and displayed only a minor drop in relative conservation score in nucleotides following the PTC. Importantly, the PTC and surrounding nucleotides displayed an overall higher conservation score relative to random exons, and this was particularly striking for PTCs in BMM. Notably, removal of the UCE-containing core splicing factor genes did not influence the conservation profile in liver or BMM (data not shown). The BMMs are characterized by a more stringent splice pattern, whereas the liver has a high degree of AS and increased PTC+ junctions expressed at low levels (Figures 2 to 4 and 6), pointing to an increased fraction of random splice byproducts in the liver. Hence, the increased PTC+ exonic conservation in BMMs compared to the liver is likely due to a higher proportion of functionally relevant PTCs in BMMs, found in various vertebrates. Moreover, the relatively high conservation score at and after the PTC suggests that these sequences may possibly harbor un-recognized *cis *elements that could assist in recognizing the PTC.

Finally, we also gauged whether PTCs associated with highly regulated PTC-inducing junctions displayed any preference for the identity of the stop codon (Figure 7b). Surprisingly, whereas the frequencies of normal canonical UAA, UAG and UGA RefSeq stop codons corresponded closely to the distribution observed in exonic regions, regulated PTCs had a marked and highly significant preference for UGA in both tissues. These findings suggest that the NMD eliciting PTCs have been under selective pressure in order to adapt to the regulatory needs of AS-NMD.

## Discussion

Here, we present the first RNA-seq study from genetically modified adult mice accompanied by a thorough comparative study of the role of NMD in two distinct murine tissues. Moreover, this is the first analysis to date of the global impact of NMD on all major classes of AS in untransformed mammalian cells.

To facilitate a detailed analysis of AS, we have generated a streamlined bioinformatic pipeline, RAINMAN, available to the scientific community, that maps RNA-seq reads to a comprehensive combinatorial exon-exon junction database to obtain maximum AS isoform information. This method allowed us to map 28% of all mappable reads to junctions (all samples combined; Figure 1) with a total discovery of 150,000 unique junctions, thus giving us high AS information. The junction data are further processed in the pipeline to predict NMD susceptibility and thus coding potential with high accuracy at single junction resolution. Finally, seven major splice isoform classes are inferred and processed for multiple comparison purposes.

### Loss of NMD leads to a pronounced deregulation of alternative splicing

Using this pipeline, we have shown that ablating *Upf2*, and by inference NMD, impacts on several layers of AS in both BMMs and liver. By examining genes that harbor the exact same spliced junctions predicted to elicit a PTC in both tissues, our data confirmed that genes involved in mRNA processing are highly overrepresented among common NMD targets, as previously shown (Table S3 in Additional file 4). From our data, we estimate that close to one-third of all junction events supporting AS result in PTC+ and NMD-sensitive transcripts in the liver (Figure 3b), which is in agreement with earlier predictions [4]. Previous computational analyses of dbEST and SWISS-PROT have found that 8 to 12% of human genes are predicted to be targets of NMD [4,40], but these repositories inherently underestimate the true number of NMD-sensitive isoforms. Our data demonstrated that 16% and 28% of expressed genes in BMM and liver, respectively, were predicted to undergo NMD (upregulated more than two-fold in KO) of one or more isoforms. These numbers expand upon and underscore the global importance of NMD and in particular AS-NMD. Nearly all detected PTC+ events could be attributed to AS, and we found that approximately 50% of all AS in the KO was in fact predicted to elicit a PTC (Figure 3a), with the liver as the tissue with the most AS (Figure 3b). This complements previous findings showing that the liver has one of the highest levels of AS compared to other organs [2,34]. The high level of AS in the liver was also reflected in the fraction of low-level PTC+ junctions (Figure 6), most likely consisting of splice errors. Thus, our data make a strong case for NMD playing an important role in removing 'splicing' noise also in untransformed mammalian tissues.

In contrast to the splice error-generated PTC+ isoforms, our analysis of highly regulated splice classes (ΔPSI > 20% or ΔPSI < -20%), revealed that approximately 40% of all highly skipped exons were degraded by NMD (Table 1). These ES events were further subdivided into SES and MES events. Whereas three-quarters of all ES events in BMMs were SES, the proportion of SES and MES in liver was approximately equal. We speculate that the increased MES proportion reflects the more promiscuous splicing pattern in the liver, thus leading to novel AS between exons not normally spliced together.

Importantly, our analysis of conservation patterns in SES revealed a high conservation score of NMD-regulated skipping exons and their flanking introns compared to that of unregulated events. This suggests that events deregulated in the absence of NMD are under tight control in normal NMD-proficient cells and that AS-NMD plays a crucial role in controlling the expression of a vast number of genes.

### The splice isoform inference analysis expands the repertoire of NMD targets

From our splice isoform inference, we found a high proportion of PTC+ events in most classes with the notable exception of MXEs (ALE events were not included in PTC classification). Due to the regulatory nature of MXEs, in which two coding exons compete for the selective inclusion, it is not surprising that this group is underrepresented in the PTC+ events. One interesting example was Pkm2, in which a well-characterized MXE event either dictates whether the adult form (M1 isoform containing exon 9) or the embryonic isoform (M2 containing exon 10) is produced. In many tumors, the embryonic M2 isoform is switched on, leading to increased aerobic glycolysis [33]. However, we found that ablating UPF2, and by inference NMD, led to inclusion of both mutually exclusive exons with a resulting downstream PTC (Figure 4b, d), suggesting an interesting function for NMD in controlling MXE. We also provide the first report of a surprisingly high frequency of PTC+ A5SS and A3SS events, suggesting that the alternative usage of splice acceptor or donor sites is often utilized to regulate expression by AS-NMD. Another possibility could be that these events are more error-prone - for example, by the use of a strong and weak splice site (the alternative splice site), leading to a less stringent AS than between two strong splice sites such as SES. Nevertheless, our study significantly expands the known repertoire of NMD targets in all classes of AS, many previously uninvestigated on a global scale.

### Common and tissue-specific functions of NMD

The impact of NMD on transcriptome composition has up to now only been studied in whole organisms or individual cell lines, which has therefore precluded any insights into conserved versus tissue-specific functions of NMD. Focusing first on core NMD targets, our stringent comparative analysis found 50 genes with a common highly upregulated PTC upon exon exclusion in BMMs and liver (Table S8 in Additional file 9). Almost all of these genes were upregulated in both tissues, suggesting a functional role for AS-NMD in regulating the abundance of transcripts with full coding potential from these genes (see below). Several of the known splicing factors previously shown to utilize AS-NMD in an autoregulatory loop, such as *Ptbp1*, demonstrated PTC upon exclusion in both tissues, but below ΔPSI of 20% in BMMs. Besides SR proteins and hnRNPs, most of the genes found upregulated are unknown targets for AS-NMD, such as *Soat2*/and *Acat2*. The latter gene is involved in esterification of cholesterol, and is known to be regulated in a tissue-specific manner, with a particularly high expression level in liver and intestine and to a lesser extent in macrophages [41]. Here, we found that this gene was upregulated 8.4-fold in BMMs and 4.2-fold in liver upon muting NMD, suggesting an important regulatory function for AS-NMD in the synthesis of cholesterol esters.

We found 26 genes with a common PTC upon inclusion event (ΔPSI > 20%) in both BMM and liver KO (Table S7 in Additional file 8). An example of a gene with a PTC upon inclusion isoform is *Nktr*, which is exclusively expressed in and required for natural killer (NK) cells. We found that inhibiting NMD caused a high upregulation of a PTC+ isoform and a concomitant 2.4- to 3.9-fold upregulation at the gene level. It has been shown that aberrant isoforms of the *Nktr *gene are present in cells not expressing *Nktr *[42]. Here, we show that NMD mutes *Nktr *in BMMs and liver (and also in NMD ablated transformed MEFs [32]), suggesting that AS-NMD serves an important regulatory function in regulating the functional expression of this NK cell-specific protein. Interestingly, *Nktr *is involved in NK cell activity but contains RS repeats and a cyclophilin-domain found in several splicing factors, and this could confer on the protein properties sufficient to facilitate regulation of its own expression through AS-NMD. Thus, it could be that several other proteins not directly involved in splicing have gained RNA-binding properties that would allow them to autoregulate their own synthesis by AS-NMD.

Apart from the core NMD targets, our analysis also yielded the first insights into potential tissue-specific effects of NMD. Through GO analysis of parent genes of PTC+ junctions that were exclusively upregulated in either the BMM or liver datasets, we could show a tissue-specific role for NMD in the regulation of G-protein-coupled receptors and mitochondria function, respectively. Interestingly, these GO classes mirror the main biological functions of monocytes/macrophages and liver tissue, that is, in immunological reactions and energy metabolism, respectively, and we predict that NMD-dependent transcriptome analysis in other organs would uncover tissue-specific NMD targets in pathways of particular importance for the organ in question.

### NMD controls the expression of a network of splicing factors

In contrast to the well-characterized role of AS-NMD in the autoregulation of individual splicing factors, the global consequences of its disruption on broad splicing patterns have not been studied in detail. We were therefore intrigued by finding that approximately 50% of the upregulated AS events in the NMD-deficient tissues were devoid of PTCs, suggesting that they were indirect targets of NMD and that the importance of NMD in AS extends well beyond AS-NMD.

Using conventional microarray-based gene expression analysis, we have previously shown that several splice factors were upregulated in both UPF2-deficient BMMs and liver [30,43]. However, these studies could not discriminate between canonical mRNA isoforms and stabilized PTC+ isoforms, which is important as many of the latter would be predicted to encode truncated and thereby (most likely) non-functional proteins. Using our RNA-seq data, and correcting for the presence of the PTC+ isoforms, we can now show deregulated expression of the canonical mRNA isoform (encoding the full-length protein) for a prominent number of splice factors, a finding that was also corroborated by changes in protein levels for two of these. These findings suggest that the prominent deregulation of PTC- containing events, at least in part, can be explained by changes in the protein levels of splicing factors. This is further supported by the observation that the intronic regions surrounding deregulated PTC- exons are highly conserved, suggesting that they are subjected to splice factor-mediated regulation.

Our data therefore extend the importance of NMD to the regulation of PTC-deficient mRNA isoforms through the deregulation of splice factor levels. The mechanisms(s) by which this occurs will be the subject of future studies. They also provide a clue as to why the liver is more affected by the loss of NMD than BMMs, as the former organ displays much higher levels of AS and is therefore predicted to be more sensitive to alterations in splicing factor levels.

Finally, and most importantly, these findings expand the functional impact of NMD on transcriptome behavior to also include substantial indirect effects on PTC- splicing events and highlights the crucial importance of this pathway as a gatekeeper of transcriptome integrity.

### A role for the stop codon and its flanking regions in regulating NMD

The importance of PTC identity and its flanking regions in the regulation of NMD is a relatively unexplored area and has not previously been addressed on a global scale. Here we were able to show that regions flanking regulated PTCs display a divergent and increased level of conservation when compared to both canonical stop codons and exonic regions, suggesting that they have been under selective pressure. Similarly, the distribution of regulated PTCs was markedly different from that of normal termination events with a strong preference for the NMD machinery to use UGA. This is interesting in light of recent findings showing that sequences (including stop codons) having positive impact on stop codon read-through lead to a reduction of NMD through the removal of UPF1 from the 3' UTR through a translation-dependent mechanism [44]. These findings may suggest that the PTC and its surrounding sequences have been under selective pressure to optimize NMD efficiency in a process involving translational read-through, which in turn may be used by the cell for regulatory purposes.

## Conclusions

By developing and applying a robust bioinformatic pipeline mediating a high-resolution study of AS and its dynamics, this whole-transcriptome analysis of two NMD-deficient primary mouse tissues provides a comprehensive quantification of the impact of NMD on untransformed mammalian transcriptomes, providing crucial novel insights into its role in both core and tissue-specific regulation of gene expression, significantly extending the importance of NMD from an mRNA quality pathway to a regulator of several layers of gene expression. Thus, in addition to removing low levels of splicing 'errors', and destabilizing targets of AS-NMD, our analysis reveals the potentially crucial importance of NMD in maintaining splicing homeostasis. In particular, the observed deregulation of full-length splicing regulators suggests that the NMD pathway controls their expression in a manner distinct from direct AS-NMD, and that their deregulation is an important contributor to the global deregulation of AS that we observe in NMD-deficient tissues.

Finally, we have provided a reference catalogue of NMD-regulated AS events as well as an open source tool, RAINMAN, facilitating the process of splice isoform inference and PTC prediction from RNA-seq reads. Future transcriptome studies in other organs and organisms will further reveal the impact of NMD on splicing patterns and how this pathway modulates biological read-out in a tissue- and pathway-specific manner.

## Materials and methods

### Mice

For the selective deletion of UPF2 in liver and BMM we used our conditional floxed *Upf2 *line and the *LysMCre *and *Mx1Cre *driver lines as described previously [7]. *Upf2 *is recombined during macrophage differentiation, and the mature BMMs are devoid of any functional UPF2 protein as well as NMD activity. To rescue the *Upf2^fl/fl^; Mx1Cre *from the previously reported hematopoietic lethality, *Upf2^fl/fl^; Mx1Cre *and *Upf2^fl/fl ^*were transplanted with wild-type bone marrow cells prior to poly-I:C injection. As described previously, liver was harvested 3 weeks after *Upf2 *recombination to avoid any indirect effects from the poly-I:C treatment [30]. All mouse work was performed according to national and international guidelines and approved by the Danish Animal Ethical Committee. This study was approved by the review board at the Faculty of Health, University of Copenhagen (LT-P0658).

### cDNA synthesis for RNA-seq

Total RNA was harvested in Trizol (Invitrogen; Carlsbad, CA, USA from *Upf2^fl/fl^; LysMCre *and *Upf2^fl/fl^*-derived male BMMs, grown as previously described [7]. Briefly, bone marrow cells were grown *in vitro *in the presence of macrophage colony-stimulating factor-conditioned medium for 7 days, giving essentially a 100% pure macrophage population. Total RNA from liver was harvested in Trizol 21 days after injection of poly I:C from *Upf2^fl/fl^; Mx1Cre *and *Upf2^fl/fl^; Mx1Cre*. We pooled 50 μg RNA from each of three age-matched males to get a total of 150 μg RNA, which was subjected to two rounds of mRNA purification by hybridizing to oligo(dT) beads (Dynabeads mRNA purification kit, Invitrogen). The resulting mRNA (1 μg) was then used as template to prepare cDNA. Double-stranded cDNA was essentially prepared as described by the manufacturer (Superscript Double-Stranded cDNA Synthesis kit, Invitrogen), using 10 μM random hexamers to prime first strand synthesis. Finally, the double-stranded cDNA was purified using QiaQuick PCR columns (Qiagen, Hilden, Germany) followed by phenol-chloroform extraction. The quality of the cDNA was verified using a Bioanalyzer.

Library preparation for cDNA sequencing was performed essentially as for the whole genome DNA library construction (DNA sample prep kit, Illumina; see Supplementary Methods in Additional file 1). Sequencing was performed on an Illumina Genome Analyzer II flowcell, generating 75 bp single-end reads. RNA-seq data have been submitted to the NCBI Short Read Archive database with accession number GSE26561.

### RT-PCR and western blotting

Total RNA was purified using Trizol and 0.5 μg RNA was used to synthesize single-stranded cDNA with oligo d(T) primers, using a ProtoScript M-MULV First-Strand Synthesis kit (New England Biolabs, Ipswich, MA, USA) as described by the manufacturer. The cDNA was used in standard PCR reactions using Taq Polymerase (Invitrogen), with primers specific for the exons flanking the alternative exon (see Table S9 in Additional file 12 for a list of primers). For protein analysis, snap-frozen tissue was dissected and lysed in ice-cold RIPA buffer supplemented with proteinase inhibitors and separated by SDS-PAGE and subjected to western blotting. Antibodies used: affinity-purified rabbit α-UPF2 (kind gift from Dr Jens Lykke-Andersen); rabbit α-mAb104 serum (pan-SR protein antibody; kind gift from Dr Javier Caceres); mouse α-β actin (ab6276, Abcam, Cambridge, UK). Development of α-mAb104 immunoblotted liver protein lysate required different exposure times depending on the molecular weight band, and was thus treated individually for the different protein bands.

### RAINMAN overview

Figure S1B in Additional file 2 outlines the sequential bioinformatic processing steps making up the RAINMAN pipeline utilized in this study. Our PTC and splice event detection and quantification pipeline is available as a series of Python and R scripts, readily scalable and customizable for use with RNA-seq short reads of any length. First, an index is generated, consisting of the relevant genome assembly combined with a combinatorial exon-exon junction database, as described in detail in the Supplementary Methods in Additional file 1. Reads are mapped to this index (using the Bowtie/TopHat [31,39] mappers as default) and unmapped reads are truncated to rescue reads spanning more than two exons. Gene expression is calculated by the RPKM metric (analogous to fragments per kb of exon per million fragments mapped (FPKM)) using the Cufflinks package [45] and upper quartile normalization, and junction expression levels are calculated by quantifying reads spanning exon-exon boundaries, likewise using the RPKM metric, and are (optionally) normalized using "Trimmed mean of M component" (TMM) normalization [46]. Finally, junctions are processed by PTC and splice event detection algorithms, and all data are output, allowing the researcher to visualize results in UCSC Genome Browser and spreadsheet software. See Supplementary Methods in Additional file 1 for a detailed description of RAINMAN and Table S6 in Additional file 6 for splice detection validation. RAINMAN scripts, documentation and complete junction and gene expression lists are available online [47].

### Statistical methods

The statistical package R was used to calculate Chi-square and Kolmogorov-Smirnov tests.

### Database accession

RNA-seq data are available at the NCBI Short Read Archive database (accession number GSE26561). RAINMAN scripts, documentation and complete junction and gene expression lists are available online [47].

## Abbreviations

A3SS: alternative 3' splice site; A5SS: alternative 5' splice site; AFE: alternative first exon; ALE: alternative last exon; AS: alternative splicing; AS-NMD: alternative splicing coupled to nonsense-mediated mRNA decay; BMM: bone marrow-derived macrophage; bp: base pair; ES: exon skipping; EST: expressed sequence tag; GO: Gene Ontology; hnRNP: heterogeneous nuclear ribonucleoprotein; KO: knock-out; MEF: mouse embryonic fibroblast; MES: multiple exon skipping; MXE: mutually exclusive exon; NK: natural killer; NMD: nonsense-mediated mRNA decay; PCR: polymerase chain reaction; PSI: percent spliced in; PTC: premature termination codon; PTC-: PTC absence; PTC+: PTC containing; RAINMAN: RnAseq-based Isoform detection and NMd ANalysis pipeline; RNA-seq: next generation RNA sequencing; RPKM: reads per kb of gene model per million mapped reads; SES: single exon skipping; SR: serine/arginine-rich; UCE: ultraconserved element; UTR: untranslated region; WT: wild type.

## Competing interests

The authors declare that they have no competing or conflicting interests.

## Authors' contributions

JoWe carried out experiments, drafted the manuscript and conceived the study. JoWa carried out the bioinformatic analysis and helped to draft the manuscript. GT, JZ, JuWa, and KK facilitated sequencing. ID and JSJ assisted with experimental work. AK participated in study design. BP helped to draft the manuscript and conceived the study. All authors have read and approved the manuscript for publication.

## Supplementary Material

Additional file 1 **Supplementary Information and Supplementary Tables S1, S2 and S5**. Supplementary Materials, Methods and References. Supplementary Table S1: number of mapped junctions contributed uniquely by the combinatorial database (Comb DB only) or TopHat (TopHat only) and the number of mapped junctions detected by both the combinatorial database and TopHat (Both). Supplementary Table S2: the contribution of TopHat to the number of junctions predicted to generate a PTC versus all junctions (minimum of three reads per junction). Supplementary Table S5: deregulation of core splice factors. Gene FC indicates the change in mRNA levels for all the isoforms for the particular gene between KO and WT. Click here for file

Additional file 2 **Supplementary Figure S1**. UCSC Genome browser output of Pion gene and schematic of the RAINMAN pipeline with steps for mapping and processing of reads. Click here for file

Additional file 3 **Supplementary Figure S2**. Histogram showing distance from normal stop codon to the 3' end of RefSeq genes with stops in final exon, and distances to nearest downstream exon-exon junction for genes with stop codons in the second to last exon. Click here for file

Additional file 4 **Supplementary Table S3**. Reads per unique junction statistics for all samples, split into junctions discovered by mapping to the combinatorial database versus junctions discovered by TopHat. Click here for file

Additional file 5 **Supplementary Table S4**. Table with Gene Ontology terms associated with genes containing upregulated PTC+ junctions that are unique for Upf2 KO liver or BMM. Click here for file

Additional file 6 **Supplementary Table S6**. Results from validation by manual inspection of output from isoform class inference. Click here for file

Additional file 7 **Supplementary Figure S3**. Validation of expression change inference and isoform inference. Click here for file

Additional file 8 **Supplementary Table S7**. PTC upon inclusion isoforms (SES) upregulated in both Upf2 KO liver and BMM (ΔPSI > 20%). Click here for file

Additional file 9 **Supplementary Table S8**. PTC upon exclusion isoforms (SES) upregulated in both Upf2 KO liver and BMM (ΔPSI < -20%). Click here for file

Additional file 10 **Supplementary Figure S4**. Mean per position phastCon conservation score around single exon skipping events for BMMs. Numbers on x-axis indicate nucleotide intervals - 25 and 75 nucleotides for exons and flanking introns, respectively. Click here for file

Additional file 11 **Supplementary Figure S5**. Conservation around upregulated PTCs, with mean per-position phastCon scores centered on the PTC for upregulated junctions in liver and BMMs. Click here for file

Additional file 12 **Supplementary Table S9**. List of primers used in RT-PCR validation of splicing events. Click here for file
