# Supplementary material for: Mammalian tissues defective in nonsense-mediated mRNA decay display highly aberrant splicing patterns
Source: Genome Biol. 2012 May 24;13(5):R35. doi: 10.1186/gb-2012-13-5-r35 (PMC3446288; doi:10.1186/gb-2012-13-5-r35)
Supplement: Additional file 1 — Supplementary Information and Supplementary Tables S1, S2 and S5. Supplementary Materials, Methods and References. Supplementary Table S1: number of mapped junctions contributed uniquely by the combinatorial database (Comb DB only) or TopHat (TopHat only) and the number of mapped junctions detected by both the combinatorial database and TopHat (Both). Supplementary Table S2: the contribution of TopHat to the number of junctions predicted to generate a PTC versus all junctions (minimum of three reads per junction). Supplementary Table S5: deregulation of core splice factors. Gene FC indicates the change in mRNA levels for all the isoforms for the particular gene between KO and WT. [file gb-2012-13-5-r35-S1.DOC]

Supplemental Material:

**Inventory:**

Supplementary figure S1: Weischenfeldt_FigS1.eps

Supplementary figure S2: Weischenfeldt_FigS2.eps

Supplementary figure S3: Weischenfeldt_FigS3.eps

Supplementary figure S4: Weischenfeldt_FigS4.eps

Supplementary figure S5: Weischenfeldt_FigS5.eps

Supplementary table S1: included in this document.

Supplementary table S2: included in this document.

Supplementary table S3: Weischenfeldt_et_al_TableS3.xls

Supplementary table S4: Weischenfeldt_et_al_TableS4.xls

Supplementary table S5: included in this document.

Supplementary table S6: Weischenfeldt_et_al_TableS6.xls

Supplementary table S7: Weischenfeldt_et_al_TableS7.xls

Supplementary table S8: Weischenfeldt_et_al_TableS8.xls

Supplementary table S9: Weischenfeldt_et_al_TableS9.xls

Supplemental methods and references included in this document.

# Supplemental Figure legends

**Figure S1**

A) UCSC Genome browser output of *Pion* gene from RAINMAN. Shown are junction evidence from mapped reads (horizontal bars) and exon coverage (vertical bars) for BMM WT and KO (top) and liver WT and KO (bottom). B) Schematic of the RAINMAN pipeline with steps for mapping and processing of reads to junctions to obtain PTC detection and isoform inference. See experimental procedures for details.

**Figure S2**

A) Histogram showing distance from normal stop codon to the 3’ end of RefSeq genes with stops in final exon. B) Distances to nearest downstream exon-exon junction for genes with stop codons in the second to last exon.

**Figure S3**

Validation of expression change inference and isoform inference. A) qPCR on 18 up- or down-regulated genes in liver KO samples (R=0.975, Pearson’s correlation, median of three biological replicates), seven of which are splicing factors. B) High consistency between biological replicates, shown by low inter-genotype sample variation in qPCR levels for 8 genes in liver WT and KO tissues. C) Cloning and sequencing of full-length cDNA isoforms of *Srsf9* of the major (PTC-) isoform (blue star) and minor (PTC+) isoform (red star) from *Upf2* WT and KO liver tissues. The three cDNA species shown in the gel (top) were Sanger sequenced and contig sequences were aligned to the reference assembly using BLAT. UCSC screenshot (bottom) shows perfect alignment with the two curated RefSeq isoforms. Red-colored exon denotes the PTC+ exon.

**Figure S4**

Mean per position phastCon conservation score around single exon skipping events for BMMs. Numbers on x-axis indicate nucleotide intervals, 25 and 75 nt for exons and flanking introns, respectively.

A) Exon exclusion events upregulated in KO sample are shown. B) shows exon inclusion events upregulated in the KO sample. PTC upon exclusion/inclusion events (red), and exclusion/inclusion events not predicted to elicit a PTC (green line), unregulated skipping events (grey line). Yellow lines are scores for all mm9 RefSeq exons and 25 bp into surrounding introns. PTC- SES were significantly different from both nonregulated and PTC+ SES regions in introns (P < 2x10e-16, Komogorov-Smirnov test). Exclusion events PTC+: 122, PTC-: 88. Inclusion events PTC+: 64, PTC-: 69. Non-regulated events: 686. Mm9 RefSeq exons: 274,281. Curves represent a cubic smoothing spline fitted to data, with ~3 nt / degree of freedom.

**Figure S5**

Conservation around upregulated PTCs. Mean per-position phastCon scores are shown, centered on the PTC, for upregulated junctions in Liver and BMM. Ranges of scores do not extend into introns, and may be shorter than 100bp for individual PTCs. Normal STOPs are based on all RefSeq transcript models. Random Ensembl is based on positionally random exonic sampling from a random subset of 4,000 Ensembl exons. For upregulated junctions, a KO/WT fold change of 2 was required. BMM PTC+ positions: 884. Liver PTC+ positions: 3,091. Normal RefSeq STOP positions: 23,231.

# Supplemental Table legends

Supplementary Table S1

Number of mapped junctions contributed uniquely by the combinatorial database (Comb DB only) or TopHat (TopHat only) and the number of mapped junctions detected by both the combinatorial DB and TopHat (Both).

Supplementary Table S2

The contribution of TopHat to the number of Junctions predicted to generate a PTC vs. all junctions. Min. 3 reads per junction.

**Supplementary Table S3**

Reads per unique junctions statistics for all samples, split in junctions discovered by mapping to the combinatorial database, versus junctions discovered by TopHat. The table demonstrates comparable mapping statics for the two approaches.

**Supplementary Table S4**

Excel table with gene ontology terms associated with genes containing upregulated PTC+ junctions that are unique for *Upf2* KO liver or BMM.

**Supplementary Table S5**

Deregulation of core splice factors. Gene FC indicates the change in mRNA levels for all the isoforms for the particular gene between KO and WT.

WT and KO ratios represent the fraction of the major upregulated, PTC-eliciting splice event for each gene. Canonical Fc is a corrected value indicating the fold change for the canonical transcript predicted to encode the full-length protein, calculated using the following formula: Gene Fc*(1-KO ratio)/(1-WT ratio)

**Supplementary Table S6**

Results from validation by manual inspection of output from isoform class inference.

**Supplementary Table S7**

PTC upon inclusion isoforms (SES) upregulated in both *Upf2* KO liver and BMM (ΔPSI>20%).

**Supplementary Table S8**

PTC upon exclusion isoforms (SES) upregulated in both *Upf2* KO liver and BMM (ΔPSI<-20%).

**Supplementary Table S9**

List of primers used in RT-PCR validation of splicing events.

**Supplementary Table S**1

|  | BMM WT | BMM KO | Liver WT | Liver KO |
| --- | --- | --- | --- | --- |
| Comb. DB only | 34,131 (25%) | 34,981 (24%) | 89,890 (45%) | 106,165 (43%) |
| TopHat Only | 14,749 (11%) | 20,401 (14%) | 21,749 (11%) | 38,640 (16%) |
| Both | 86,427 (64%) | 89,714 (62%) | 88,144 (44%) | 100,463 (41%) |
| Total | 135,307 | 145,096 | 199,783 | 245,268 |

**Supplementary Table S**2

|  | BMM | Liver |
| --- | --- | --- |
| All junctions | 99,657 | 128,617 |
| PTC+ junctions | 4,047 | 14,387 |
| All Tophat junctions | 6,489 | 11,833 |
| PTC+ Tophat junctions | 1,328 | 2,888 |
| Tophat contr., all junctions | 4 % | 11 % |
| Tophat contr. PTC+ junctions | 20 % | 24 % |

**Supplementary Table** S5

| **Gene symbol** | **BMM Gene Fc** | **BMM WT ratio** | **BMM KO ratio** | **BMM canonical transcript fc** | **Liver Gene Fc** | **Liver WT ratio** | **Liver KO ratio** | **Liver canonical transcript fc** |
| --- | --- | --- | --- | --- | --- | --- | --- | --- |
| **Srsf1** | 1.02 | - | - |  | 0.93 | - | - |  |
| **Srsf2** | 0.87 | - | - |  | 1.31 | 0.00 | 0.08 | 1.20 |
| **Srsf3** | 1.71 | - | - |  | 5.11 | 0.00 | 0.16 | **4.29** |
| **Srsf4** | 1.86 | - | - |  | 3.45 | 0.00 | 0.14 | **2.97** |
| **Srsf5** | 1.17 | 0.15 | 0.16 | 1.15 | 0.74 | - | - |  |
| **Srsf6** | 1.31 | - | - |  | 1.57 | 0.00 | 0.12 | 1.38 |
| **Srsf7** | 0.82 | - | - |  | 1.46 | - | - |  |
| **Srsf8** | 1.29 | - | - |  | 1.44 | - | - |  |
| **Srsf9** | 1.10 | - | - |  | 0.98 | - | - |  |
| **Tra2b** | 1.73 | - | - |  | 3.01 | 0.75 | 0.97 | **0.36** |
| **Tra2a** | 1.27 | - | - |  | 3.02 | 0.00 | 0.32 | **2.05** |
| **Srfs11** | 1.50 | 0.80 | 0.96 | **0.30** | 2.78 | 0.67 | 0.91 | 0.76 |
| **Srsf16** | 1.91 | - | - |  | 2.93 | 0.00 | 0.30 | **2.05** |
| **Hnrnpa2b1** | 1.40 | - | - |  | 1.38 | 0.36 | 0.57 | 0.93 |
| **Hnrnpd** | 0.93 | - | - |  | 1.06 | 0.00 | 0.19 | 0.86 |
| **Hnrnpf** | 1.00 | - | - |  | 0.99 | 0.31 | 0.87 | **0.19** |
| **Hnrnph1** | 1.38 | 0.06 | 0.46 | 0.79 | 4.66 | 0.37 | 0.89 | 0.81 |
| **Hnrnph3** | 4.67 | - | - |  | 3.81 | 0.25 | 0.88 | **0.61** |
| **Hnrnpk** | 1.00 | 0.00 | 0.73 | **0.27** | 0.95 | 0.00 | 0.07 | 0.89 |
| **Hnrnpl** | 1.12 | 0.00 | 0.19 | 0.91 | 1.69 | 0.00 | 0.34 | 1.12 |
| **Hnrnpr** | 1.01 | - | - |  | 0.83 | 0.00 | 0.21 | **0.66** |
| **Hnrpdl** | 3.27 | - | - |  | 2.28 | 0.00 | 0.18 | **1.87** |
| **Ptbp1** | 0.97 | 0.01 | 0.17 | 0.81 | 1.37 | 0.10 | 0.38 | 0.95 |
| **Ptbp2** | 4.38 | 0.50 | 1.00 | **0.00** | 3.99 | 0.25 | 1.00 | **0.00** |
| **Hnrpll** | 1.46 | 0.00 | 0.32 | 0.99 | 1.66 | - | - |  |

# Supplemental Methods

**Illumina library preparation**

Illumina library preparation for cDNA sequencing was performed as described in the whole genome DNA library construction (DNA sample prep Kit, Illumina). As input, 1 ug cDNA in 50ul TE buffer was fragmented by nebulization with compressed nitrogen gas at 32psi for 9 minutes. Nebulization generated double-stranded DNA fragments with blunt ends or with 3’ or 5’ overhangs. The overhangs were converted to blunt ends by using T4 DNA polymerase and Klenow polymerase. Then an “A” base was added to the ends of double-stranded DNA using Klenow exo- (3’ to 5’ exo minus). Next, the DNA adaptors (Illumina) with a single “T” base overhang at the 3’ end was ligated to the above products, and ligation products ranging from 150 to 250bp were purified on 2% agarose gels (Qiagen Gel Extraction Kit). The adapter-modified DNA fragments were enriched by PCR with PCR primer 1.1 and 2.1 (Illumina). Separate 12-cycle reactions were used for sequencing. The concentration of the libraries was measured by absorbance at 260nm. Sequencing was performed on an Illumina Genome Analyzer II flowcell, generating 75 bp single-end reads.

**Mapping of RNA-seq reads.**

RNA-seq reads were mapped to the July 2007 NCBI / MGI mouse genome assembly (mm9), as well as a combinatorial database of exon-exon splice junctions (described below), using the Bowtie short read aligner [1] (ver. 0.11.3) and allowing for up to two mismatches in the first 68 bp. In case of multi-mapping of reads, the best hit in terms of mismatch stratum was selected. To recover reads to exons (and splice junctions for these) not included in repositories (see below), TopHat [2] (ver. 1.0.14), an aligner that map reads to junctions without reference annotation, were utilized. This approach recovered between ~15,000 (~11%) and ~39,000 (~16%) splice junctions (Table S1). Splice junctions detected only via the combinatorial database, but not by TopHat, were from ~34,000 (~25%) to ~126,000 (~43%) depending on sample (see Table S1), emphasizing the need for combining these two approaches for deep junction discovery.

To validate the accuracy of mapping, ~830,000 reads were generated, spanning 1,000 randomly selected RefSeq transcript models (exons as well as junctions) with read coverages from 1 to 10,000 reads per transcript model. The correlation coefficient (R^2^) between simulated and correctly mapped reads was ~ 0.93; 93.73% of reads were mapped, and 89.12% of reads were mapped correctly. Altering parameters such as unique hits vs. best or mapping seed length, did not improve mapping performance (data not shown). Mapped data for all samples was output to BigWig (genomic) or BED (junctions) format [3] and uploaded to the UCSC genome browser for visualization (example in Figure 1C).

RNA-seq data has been submitted to the NCBI Short Read Archive database with accession number GSE26561.

**Combinatorial splice junction database.**

Retrieving all murine exon evidence from 5 online repositories (UCSC Known Genes [4], Ensembl [5], RefSeq , GENSCAN [6] and Exoniphy) generated a comprehensive combinatorial splice junction database, essentially as performed by [7]. Exons from these data sets were combined in all possible combinations within each RefSeq gene-model, with a read/exon overlap requirement of 4 bp on each side of the junction, and generating 5,574,728 unique splice junctions. Splice junctions of consecutive exons according to RefSeq were marked as “Canonical”. Finally, databases for read lengths of 65, 55, 45 and 35 nt was created, for mapping of truncated reads (described below). Full databases are available for download at http://people.binf.ku.dk/jwaage/RAINMAN/.

**Read truncation.**

As approx. 13% of murine exons are shorter than 75 nt, several reads are expected to span more than two exons. To recover these during the mapping procedure, unmapped reads were truncated from either side in steps of 10 nt, and remapped. Reads were truncated down to 35 nt, at which length mapping ambiguity prevents further shortening. This approach recovered 781,275 reads in BMM WT (~7% of total reads), 790,321 reads in BMM KO (~6.7% of total reads), 716,923 reads in Liver WT (~3.5% of total reads), and 776,754 reads in Liver KO (~4% of total reads).

**Transcript and junction expression quantification.**

Gene expression was calculated in units of Reads Per Kilobase of exon per Million mapped reads (RPKM, analogous to FPKM for single end sequencing, “RPKM” is used in this and the main text), using the Cufflinks package [8], with standard parameters and using upper quartile normalization. Junction expression was estimated by tallying reads covering exon-exon boundaries. Longest RefSeq model per MGI mouse gene symbol were used as gene boundary. To adjust for misestimation of number of reads to a given junction due to the “sequencing real estate problem”, reads were TMM normalized using the EdgeR package of BioConductor [10] (v. 1.6.12). Normalization factors for samples were 0.998 (BMM WT), 1.002 (BMM KO), 1.188 (Liver WT), and 0.841 (Liver KO). Per-gene and per junction RPKM values were calculated based on normalized reads, and junction RPKM values were normalized to WT vs. KO gene RPKM to eliminate gene expression differences when comparing junction expression between WT and KO. Fold changes for genes and junctions were calculated as RPKM KO/ RPKM WT. Unless otherwise mentioned, junction evidence was defined as a junction with more than 2 reads to either WT or KO. Genes and junctions with a KO/WT fold change of >= 2 were called as upregulated; genes and junctions with a KO/WT fold change of <= -2 were called as downregulated. No weight was given based on overall gene expression level. To validate quantification of transcript levels by RNA-seq, we compared transcript RPKM levels for BMM WT with microarray data [11] using maximum observed probe intensity as array gene expression levels, and observed a correlation of ~ 0.63.

**PTC-detection.**

For PTC-detection, splice-junctions were processed in an iterative manner. First, exon models were associated with each splice-junction based on genomic coordinates of junction edges, using exons from repositories RefSeq, UCSC Known Genes, Ensembl, GENSCAN, EXONIPHY and TROMER. Junctions, for which one or both exons were not identified in repositories, were excluded from further analysis (2,665 junctions for BMM WT, 3,022 junctions for BMM KO, 7,343 junctions for Liver WT, and 7,768 junctions for Liver KO). For each junction, the longest compatible transcript model from either RefSeq or Ensembl was selected, in that order of priority. Adjacent exons of the chosen transcript model were concatenated on each side of the junction exons, and exons were numbered according to the selected model, with non-model exons being marked AE, “alternative exon”. The resulting putative open reading frame (ORF) was translated according to the RefSeq/Ensembl annotated translation start site, noting, if present, the distance from first STOP to nearest downstream exon-exon junction, the distance to the 3’ terminal exon-exon junction, the number of downstream exon-exon junctions, and the genomic coordinate of the STOP. Junctions producing ORFs with STOPs more than 49 nucleotides upstream of the 3’ terminal exon-exon junction were marked as PTCs. Genes with an overrepresentation of junctions generating PTCs with the same characteristics (mostly genes with early normal STOPS) were treated as false positives (BMM: 118 genes, liver: 190 genes), and untagged as PTC-generating. Junctions for genes with ambiguous or erroneous CDS information were excluded from analysis (BMM: 413 junctions, liver: 881 junctions). The robustness of the PTC detection algorithm was validated by assembling transcripts manually from genomic sequence for a random subset of junctions, testing for the given PTC reported by the algorithm; PTCs and distance-characteristics were called correctly in all tested cases. Furthermore, experimental validation by RT-PCR confirmed 35 of 36 PTC-inducing splice-events (Figure 4B and data not shown), confirming the robustness of our mapping and annotation pipeline.

**Splice isoform inference.**

To predict the most likely isoform for the purpose of splice class detection, exons were concatenated to the most compatible and, for multiple compatible models, the longest RefSeq or Ensembl model. This is based on the observation that the overwhelming majority of mapped junction reads span canonical exons (mean of samples ~90%), and on the assumption that AS events generating downstream stop codons can be treated as independent occurrences. Alternative splicing events were initially categorized in 7 different groups: single exon skipping (SES), multiple exon skipping (MES), alternative 5’ splice site (A5SS), alternative 3’ splice site (A3SS), alternative first exon (AFE), alternative last exon (ALE), and mutually exclusive exons (MXE) (Figure 4B). In the pipeline, each junction was tested for membership of these 7 classes in an iterative manner.

For skipping events, each junction was tested for containing other junctions fully (i.e. not only overlapping) within its left and right genomic coordinates.

Skipping junctions evident of only two skipped junctions, for whom corresponding evidence of only one common exon was present, was designated as SES. The exon inclusion ratio or Percent Spliced In (PSI) was calculated as

where r_skipping_ = reads to the skipping junction, and r_skipped_ = reads to skipped junctions.

For this, and all other event types, the inter-genotype ratio, termed delta Percent Spliced In (∆PSI), was calculated as PSI_KO_ - PSI_WT_. For all splicing classes, confidence intervals and p-values were calculated for PSI, essentially as done in [12]. Skipping events evident of skipping more than two exons, for which corresponding exon evidence was present, was designated as MES. The multiple exon inclusion ratio was calculated as

where *n* = number of junctions skipped, r_skipping_ = reads to the skipping junction, and r_skipped_ = reads to skipped junctions.

For identifying A5SS events, junctions sharing a common right edge genomic coordinate with the junction being tested were selected. For these junctions, corresponding exons sharing a common end coordinate were evidence for an alternative splice site, and the PSI for each event was calculated as

where r_tested_ = reads to the tested junction, and r_alternative_ = reads to the alternative junction. The same approach was applied for A3SS events, in reverse direction. Even though no discrete exonic elements are spliced in or out in A3SS/A5SS and AFE/ALE events, we have chosen to use PSI instead of “switch-ratios” for consistency, in the text denoting the percentage of the relevant isoform (PTC+/PTC-, up-/downregulated, etc.).

For identifying AFE, the left edge genomic coordinate of the junction tested was compared to a list of annotated first exons in repositories RefSeq, UCSC Known Genes or Ensembl. If matched, other junctions sharing the right edge genomic coordinates with the tested junction was chosen, and based on the left edge genomic coordinate of these, putative alternative exons were identified, but ignoring exons for which the genomic left coordinate was identical to that of the original annotated first exon, instead indicating an alternative splice event. Furthermore, no junctions upstream connecting to either the original or the alternative exon were allowed. Junctions passing these criteria were marked as AFE events, and the PSI was calculated as

where r_tested_ = reads to the tested junction, and r_alternative_ = reads to the alternative junction.

For detecting ALE, the same procedure was applied, in reverse genomic direction, and with exons marked as last in repository models.

For detecting MXEs, junctions sharing a common left edge genomic coordinate with the test junction were selected. Exon evidence matching the right edge genomic coordinate of both these junctions to exactly two exons was selected, if junctions connecting the end genomic coordinates had a common right edge genomic coordinate. Requirements for these mutually exclusive exons were no common start or end coordinates, no overlap, and no connecting junctions. Furthermore, lowering sensitivity somewhat, but to increase specificity, no other exons were allowed between mutually exclusive exons. The PSI between MXEs were calculated as

where r_leftmost MXE junc_ = reads to the leftmost MXE-junction, and r_rightmost MXE junc_ = reads to the rightmost MXE-junction.

For quantifying splice events, a ΔPSI threshold of <-20% (event exclusion) or >20% (event inclusion) was used.

**Validation of splice isoform inference pipeline:** To confirm the accuracy of the pipeline, we validated a subset of 63 genes (both random genes as well as genes known to utilize NMD-AS, including several SR and HNRP family members), and manually inspected and verified/rejected the output of the splice isoform inference pipeline (Table S6 in additional file 6). In 62/62 (100%) of genes, single or multiple exon skipping presence/absence was called correctly for both tissues. In 62/62 (100%) of genes for BMM, and 60/62 of genes (~96.7%) for liver, alternative 5’ splice site presence/absence was called correctly. In 60/62 (~96.7%) of genes for BMM and 61/62 (~98.3%) of genes for liver, alternative 3’ splice site presence/absence was called correctly. In 62/62 (100%) of genes, alternative first and alternative last exons were called correctly for both tissues. In 62/62 (100%) of genes, mutually exclusive exons were called correctly for both tissues.

**Conservation analysis of skipping events.** For conservation studies (figure 5 and 7), 30-way vertebrate phastCon scores [13] for the july 2007 NCBI / MGI mouse genome assembly (mm9) was downloaded from UCSC, using mean-per-position scores and standard error of the mean as uncertainty measure.

**Pipeline usage.** We have published full code and instructions for the pipeline described in this study, RAINMAN, at http://people.binf.ku.dk/jwaage/RAINMAN/.

# Supplemental References

1. Langmead B, Trapnell C, Pop M, Salzberg SL: **Ultrafast and memory-efficient alignment of short DNA sequences to the human genome.** In *Genome Biol*, vol. 10. pp. R25; 2009:R25.

2. Trapnell C, Pachter L, Salzberg S: **TopHat: discovering splice junctions with RNA-Seq.** *Bioinformatics* 2009.

3. Rhead B, Karolchik D, Kuhn RM, Hinrichs AS, Zweig AS, Fujita PA, Diekhans M, Smith KE, Rosenbloom KR, Raney BJ, et al: **The UCSC Genome Browser database: update 2010.** In *Nucleic Acids Res*, vol. 38. pp. D613-619; 2010:D613-619.

4. Hsu F, Kent WJ, Clawson H, Kuhn RM, Diekhans M, Haussler D: **The UCSC Known Genes.** *Bioinformatics* 2006, **22:**1036-1046.

5. Flicek P, Aken BL, Ballester B, Beal K, Bragin E, Brent S, Chen Y, Clapham P, Coates G, Fairley S, et al: **Ensembl's 10th year.** In *Nucleic Acids Res*, vol. 38. pp. D557-562; 2010:D557-562.

6. Burge C, Karlin S: **Prediction of complete gene structures in human genomic DNA.** In *J Mol Biol*, vol. 268. pp. 78-94; 1997:78-94.

7. Wang ET, Sandberg R, Luo S, Khrebtukova I, Zhang L, Mayr C, Kingsmore SF, Schroth GP, Burge CB: **Alternative isoform regulation in human tissue transcriptomes.** In *Nature*, vol. 456. pp. 470-476; 2008:470-476.

8. Trapnell C, Roberts A, Goff L, Pertea G, Kim D, Kelley DR, Pimentel H, Salzberg SL, Rinn JL, Pachter L: **Differential gene and transcript expression analysis of RNA-seq experiments with TopHat and Cufflinks.** *Nat Protoc* 2012, **7:**562-578.

9. Mortazavi A, Williams BA, Mccue K, Schaeffer L, Wold B: **Mapping and quantifying mammalian transcriptomes by RNA-Seq.** *Nat Meth* 2008, **5:**621-628.

10. Robinson MD, Oshlack A: **A scaling normalization method for differential expression analysis of RNA-seq data.** *Genome Biol* 2010, **11:**R25.

11. Weischenfeldt J, Damgaard I, Bryder D, Theilgaard-Mönch K, Thoren LA, Nielsen FC, Jacobsen SE, Nerlov C, Porse BT: **NMD is essential for hematopoietic stem and progenitor cells and for eliminating by-products of programmed DNA rearrangements.** In *Genes Dev*, vol. 22. pp. 1381-1396; 2008:1381-1396.

12. Jiang H, Wong WH: **Statistical inferences for isoform expression in RNA-Seq.** *Bioinformatics* 2009, **25:**1026-1032.

13. Siepel A, Bejerano G, Pedersen JS, Hinrichs AS, Hou M, Rosenbloom K, Clawson H, Spieth J, Hillier LW, Richards S, et al: **Evolutionarily conserved elements in vertebrate, insect, worm, and yeast genomes.** In *Genome Res*, vol. 15. pp. 1034-1050; 2005:1034-1050.
